# Anti-inflammatory effects of nicotine in obesity and ulcerative colitis

**DOI:** 10.1186/1479-5876-9-129

**Published:** 2011-08-02

**Authors:** Shaheen E Lakhan, Annette Kirchgessner

**Affiliations:** 1 Global Neuroscience Initiative Foundation, Los Angeles, CA, USA; 2 School of Health and Medical Sciences, Seton Hall University, South Orange, NJ, USA

**Keywords:** α7-nicotinic acetylcholine receptor, ulcerative colitis, enteric nervous system, pro-inflammatory cytokines

## Abstract

Cigarette smoke is a major risk factor for a number of diseases including lung cancer and respiratory infections. Paradoxically, it also contains nicotine, an anti-inflammatory alkaloid. There is increasing evidence that smokers have a lower incidence of some inflammatory diseases, including ulcerative colitis, and the protective effect involves the activation of a cholinergic anti-inflammatory pathway that requires the α7 nicotinic acetylcholine receptor (α7nAChR) on immune cells. Obesity is characterized by chronic low-grade inflammation, which contributes to insulin resistance. Nicotine significantly improves glucose homeostasis and insulin sensitivity in genetically obese and diet-induced obese mice, which is associated with suppressed adipose tissue inflammation. Inflammation that results in disruption of the epithelial barrier is a hallmark of inflammatory bowel disease, and nicotine is protective in ulcerative colitis. This article summarizes current evidence for the anti-inflammatory effects of nicotine in obesity and ulcerative colitis. Selective agonists for the α7nAChR could represent a promising pharmacological strategy for the treatment of inflammation in obesity and ulcerative colitis. Nevertheless, we should keep in mind that the anti-inflammatory effects of nicotine could be mediated via the expression of several nAChRs on a particular target cell.

## Introduction

The major addictive component of tobacco, nicotine, exerts anti-inflammatory effects in multiple cell types and has been shown to benefit various disorders in which an inflammation-related mechanism is implicated. Chronic low-grade inflammation is a key feature of obesity, which is characterized by the elevated production of pro-inflammatory cytokines by the adipose tissue itself [1-3]. Chronic and relapsing inflammation is at the core of inflammatory bowel disease (IBD), which is characterized by activation of the pro-inflammatory transcription factor nuclear factor-κB (NF-κB) [4] and increased expression of pro-inflammatory cytokines such as tumor necrosis (TNF)-α in immune cells in the mucosa of IBD patients [5,6]. Nicotine has been proven effective in reducing obesity-related inflammation and insulin resistance [7] and attenuating inflammation and improving gut function in patients with active colitis [8]. In fact, ulcerative colitis patients with a history of smoking usually acquire their disease after they have stopped smoking [9-11]. Patients who smoke intermittently often experience an improvement in their colitis symptoms during the periods when they smoke [9,12]. Therefore the development of drugs designed to suppress the aberrant inflammatory response in obesity and ulcerative colitis may be of significant help in giving relief to patients.

Recent studies suggest that the parasympathetic nervous system, in particular the efferent vagus nerve, regulates immune responses via the peripheral release of acetylcholine (ACh) [13,14]. Activation of the "cholinergic anti-inflammatory pathway" inhibits NF-κB signaling through the α7 nicotinic acetylcholine receptor (nAChR) on immune cells such as macrophages [13,15,16] or bone marrow-derived dendritic cells [17]. Thus, the cholinergic anti-inflammatory pathway could be exploited to suppress inflammation in obesity and gastrointestinal (GI) dysfunction. This article will discuss recent advances in understanding the anti-inflammatory effects of nicotine in obesity and gut dysfunction, including ulcerative colitis.

### Nicotine suppresses the production of pro-inflammatory cytokines

There is no doubt that the net effect of cigarette smoking is pro-inflammatory primarily as a result of increased oxidative stress, which occurs when the amount of reactive oxygen species (ROS) generated in cells exceeds the capacity of normal detoxification systems [18,19]. Oxidative stress is one potential explanation for the enhanced DNA breaks in smokers [20]. Thus, it has implications for understanding the mechanisms by which smoking induces organ damage. There is overwhelming medical and scientific consensus that cigarette smoking causes lung cancer, heart disease, emphysema, and other serious diseases in smokers. Cigarette smoke contains molecules that act as potent carcinogens (e.g., benzo[a]pyrene), as well as a large amount of ROS forming substances such as catechol or hydroquinone. However, nicotine, while being the addictive agent, is often viewed as the least harmful of these compounds. In fact, nicotine exhibits anti-inflammatory properties in many systems [15,16,21,22].

Among the earliest findings in support of the anti-inflammatory potential of nicotine was the observation that nicotine altered the capacity of cells to respond to the pro-inflammatory cytokine TNF-α [23] or inhibited the release of this cytokine from the immune cell [21]. The vagus nerve can restrain serum TNF levels, and prevents septic shock and organ damage [24]. Since ACh is the principal neurotransmitter of the vagus nerve, preliminary studies analyzed the potential of cholinergic agonists to prevent TNF production in immune cells [25]. These studies collectively defined an interaction described as the "cholinergic anti-inflammatory pathway" [21,22]. As defined in these studies, the anti-inflammatory properties of nicotine are generally restricted to α7nAChR function and require ACh release from vagal efferents [21].

Cytokines are low-molecular-weight proteins released during activation of the inflammatory cascade, which after binding to specific receptors affect immune cell differentiation, proliferation, and activity. In general, cytokines can be divided into those with predominantly pro-inflammatory actions and those with anti-inflammatory actions. Pro-inflammatory cytokines include TNF-α, interleukin (IL)-1β, IL-6, and IL-8. TNF-α is a pleiotropic cytokine involved in many of the physiological responses to infection, trauma, and cancer. In addition, it has been strongly implicated as a mediator of sepsis and studies of sepsis have shown elevated circulating levels of this cytokine [26]. Anti-inflammatory cytokines include IL1 receptor antagonist, IL-10, IL-13, and TNF-binding proteins 1 and 2 (for review see [27]).

ACh and nicotine inhibit TNF-α and NF-κB production from lipopolysaccharide (LPS)-stimulated human macrophages and splenocytes [24,28]. Both the vagus nerve and nicotine exert their inhibitory effects through the activation of Jak2 and STAT3 [15] and the anti-inflammatory action of nicotine is mediated by tristetrapolin (TTP) [29], an adenylate uridylate- rich element binding protein that promotes the degradation of a number of inflammatory mediators including TNF-α Nicotine-activated STAT3 signaling induces the expression of TTP in macrophages and, in turn, TTP plays a key role in nicotine-induced anti-inflammatory effect through destabilization of TNF-α transcripts. Since an excess of TNF-α is involved in many inflammatory diseases, the inhibition of TNF-α production through the modulation of nicotine-STAT3-TTP signaling pathway may have wide-ranging clinical implications. Interestingly, TTP-knockout mice develop severe inflammatory arthritis, autoimmune dysfunction, and myeloid hyperplasia, demonstrating the importance of TTP in limiting the inflammatory response [30].

ACh and nicotine also reduce the concentration of high mobility group box 1 (HMGB1) protein production by macrophages in sepsis patients [31]. HMGB1, a nucleosome protein that acts as a pro-inflammatory cytokine, stimulates other pro-inflammatory cytokines (TNF-α, IL-1β, and IL-8) and promotes epithelial cell permeability [31]. Treatment with nicotine attenuated serum HMGB1 levels, decreased the clinical signs of sepsis, provided significant protection against death and improved survival in "established" sepsis [31]. Additionally, nicotine treatment was not started until 24 h after the induction of lethal peritonitis in mice indicating that the cholinergic anti-inflammatory pathway can modulate the inflammatory response even in established sepsis [26].

### The cholinergic anti-inflammatory pathway

**Figure 1:**
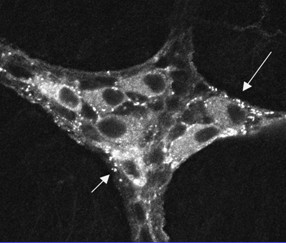
**Immunohistochemical localization of nicotinic acetylcholine receptors (nAChRs) in the guinea pig enteric nervous system**. Confocal image of a whole mount preparation of the myenteric plexus of the stomach stained using monoclonal antibody mAb35, which recognizes alpha bungarotoxin-insensitive nAChRs. Note the punctate staining around neuronal cell bodies. Reprinted from Wiley-Liss, Inc: *The Journal of Comparative Neurology *390(4): 497-514 Copyright 1998 [32].

In the GI tract, the vagus nerve regulates motility and digestive function via the activation of nAChRs classically found on enteric neurons (See Figure 1; [32]). However, non-neuronal cells, including immune cells throughout the body also express nAChRs where they contribute to diverse physiological processes including immunomodulation [17].

In general, there are two major nAChR subtypes that are composed of either homomeric subunits (e.g., α7nAChR) or combinations of alpha (α) and beta (β) subunits, and it is the final subunit configuration that imparts significant functional and pharmacological differences among these receptors (for review see [33]). Neuronal nAChRs are composed of α2-α9 and β2-β4 subunits and are divided into two types. The first type is composed of a heteromeric pentamer of α2-α6 and β2-β4 and does not bind α-bungarotoxin (BTX). The second type is composed of a homomeric pentamer of α7-α9 and can bind αBTX. The α7nAChR subunit exhibits remarkably high Ca^2+ ^permeability and thus plays an important role in Ca^2+^-dependent events, such as neurotransmitter release, cell survival and apoptosis. The expression of α7nAChR by macrophages and other immune cells suggests that it also plays a role in regulating tissue inflammation. In fact, α7nAChR is essential in mediating the anti-inflammatory effect of ACh [16].

The cholinergic anti-inflammatory pathway is a brain-to-immune mechanism that regulates inflammatory responses via α7-nAChR-dependent, vagus nerve signaling. Studies by Borovikova et al. demonstrated the potency of the vagus nerve to inhibit TNF-α production by macrophages after systemic endotoxin [13]. Peritoneal and peripheral blood mononuclear cell-derived macrophages express α7-nAChRs and vagal nerve stimulation or exogenous ACh has been shown to inhibit NF-κB transcriptional activity and pro-inflammatory cytokine production [16,31]. Studies indicate that ACh post-transcriptionally suppresses TNF synthesis and inhibits the release of IL-1β, IL-6, and IL-8 without preventing the release of the anti-inflammatory cytokine IL-10 [13]. In addition, electrical vagal nerve stimulation has been shown to ameliorate disease in animal models of inflammatory conditions including sepsis [13], ischemia reperfusion [34], hemorrhage [35] and postoperative ileus [15]. Thus, the production of pro-inflammatory cytokines from peripheral macrophages can be attenuated by vagal activity such that activation of this systemic "cholinergic anti-inflammatory pathway" improves survival during experimental sepsis [31,36]. In contrast, chemical as well as surgical blockade of vagus nerve signaling significantly worsened colitis and enhanced colonic inflammatory mediators in two experimental models of colitis [37,38], an effect that was counteracted by nicotine administration.

Additional evidence supporting the role of the vagus nerve in modulating the inflammatory response comes from studies of rats subjected to cecal ligation and puncture (CLP, a model of polymicrobial sepsis) where electrical stimulation of the efferent vagus nerve significantly decreased serum TNF-α production, hepatic TNF-α synthesis, and prevented the development of CLP-induced hypotension. In contrast, bilateral cervical vagotomy led to substantially increased serum and hepatic TNF-α levels and accelerated the development of shock [39].

Naturally occurring CD4(+)CD25(+) regulatory T cells (Tregs) are essential for the active suppression of autoimmunity, and Tregs from naïve C57BL/6J mice express α7-nAChR [40]. Moreover, nicotine via its action on α7nAChR seems to be a critical regulator for the immunosuppressive function of CD4(+)CD25(+) Tregs in mice [40]. Furthermore, nicotine reduced NF-κB-mediated transcription as measured by IL-2 and IκB transcription [41]. Together, these results suggest a "direct" link between the vagus nerve and immune cells, where ACh released by the vagus nerves activates a7nAChR on immune cells to inhibit cytokine production.

However, recent studies have shown that the spleen is a major source of inflammatory cytokines involved in the initiation of systemic inflammation [24] and that the vagus nerve can control systemic inflammation by inhibiting cytokine production in the spleen [24]. In fact, splenectomy prevents the anti-inflammatory potential of the vagus nerve. Since the vagus nerve does not innervate the spleen but terminates in the celiac-mesenteric ganglia [42], these results were surprising. Recent findings indicate that ACh released by the vagus nerve in the celiac-mesenteric ganglia activates postsynaptic α7nAChR of the splenic nerve, leading to the release of norepinephrine in the spleen [43]. Splenic norepinephrine can inhibit cytokine production from macrophages via β-adrenergic receptors [33]. Thus, both the vagus nerve and α7nAChR agonists require the splenic nerve to control systemic inflammation in sepsis. Moreover, both the parasympathetic vagus nerve and the sympathetic splenic nerve can team together and coordinate to control systemic inflammation in life threatening conditions such as sepsis.

Cholinergic signaling to the spleen also plays an important role in modulating leukocyte migration during inflammation. Endothelial cells express the α7nAChR, and pharmacologic stimulation of this receptor reduces both chemokine production and adhesion molecule expression by endothelium [44]. However, the endothelium is not directly innervated by the vagus nerve. Recent studies demonstrate that cholinergic signaling to the spleen regulates leukocyte migration to sites of tissue inflammation by reducing adhesion molecule expression [45]. Thus, the spleen is a critical interface between the cholinergic anti-inflammatory pathway and the system regulation of immune cell trafficking and the cholinergic regulation of neutrophil migration is mediated, in part, through modulation of CD11b expression on the surface of neutrophils [45]. Vagus nerve stimulation significantly attenuates neutrophil surface CD11b surface expression levels only in the presence of an intact and innervated spleen. Activating this mechanism through direct stimulation of the endogenous vagus nerve pathway to the spleen (via splenic innervation) or through administration of pharmacological cholinergic agonists (which act through the spleen) may have important therapeutic potential to inhibit excessive and deleterious neutrophil migration into inflamed or infected tissues [45].

### Nicotine ameliorates obesity-induced inflammation and insulin resistance

The World Health Organization has estimated that by 2015 approximately 2.3 billion adults will be overweight and more than 700 million obese [46]. The increase in obesity is associated with corresponding increases in type 2 diabetes, hypertension, cardiovascular disease and cancer [47]. Obesity is also associated with an increased incidence of gastrointestinal (GI) disorders [48] suggesting effects on the enteric nervous system (ENS), which controls virtually all gut functions (for review see [49]).

The appetite-suppressing effect of tobacco is well established and a major driver of smoking behavior [50]. A negative correlation among smoking, body weight, and caloric intake has been well demonstrated across species [51-53]. Mice exposed to three cigarettes, three times a day for 4 days displayed a marked decrease in food intake and body weight [52]. Animals exposed to 4 weeks of cigarette smoke had reduced food intake, body weight gain, fat mass, as well as plasma leptin concentration relative to control mice whereas equivalent food restriction only decreased body weight [54]. Moreover, potential weight gain on smoking cessation may deter people from quitting [51,52,55-57]. Such individuals should be made aware that smoking is not an efficient way to control body weight. Although the mechanisms of appetite regulation by smoking are unknown, hypothalamic energy balance circuits were disturbed by cigarette smoke exposure as evidenced by the altered neuropeptide Y (NPY) concentration in the hypothalamic paraventricular nucleus, suggesting NPY signaling is involved in the appetite-suppressive effects of cigarette smoking [54].

Nicotine, the principal addictive constituent of tobacco, has been shown to suppress appetite and attenuates obesity in many studies, but the underlying mechanism is not clear. Nicotine receptors are highly expressed in the hypothalamus and medulla, in nuclei that play a significant role in appetite regulation. Activation of hypothalamic α3β4 nAChRs led to the activation of anorexigenic pro-opiomelanocortin (POMC) neurons in the arcuate nucleus and subsequent stimulation of melanocortin 4 receptors, which were critical for the nicotine-induced decrease in food intake in mice [58]. Nicotine inhibited excitatory synaptic activity recorded in NPY, but not POMC neurons and also excited the arcuate nucleus hypocretin/orexin neurons that enhance cognitive arousal, but the responses were smaller than in POMC neurons [59]. Increased NPY expression in food-restricted rats was inhibited by nicotine administration [60] and hypothalamic NPY Y1 receptor density was reduced by chronic nicotine treatment [61]. Together, these findings indicate that nicotine has a number of actions on hypothalamic neurons that could contribute to the reduced food intake and weight loss associated with smoking.

Low-grade inflammation is a key feature of obesity and links obesity to insulin resistance, impaired glucose tolerance and even diabetes. Features of obesity-induced inflammation include increased production of pro-inflammatory cytokines, including TNF-α and IL-6 by white adipose tissue (WAT), and the activation of a network of pro-inflammatory signaling pathways, including the c-Jun NH_2_-terminal kinase (JNK) and inhibitor of NF-κB kinase β (IKKβ), which may have local effects on WAT physiology but also systemic effects on other organs [62].

Recent data indicate that obese WAT is infiltrated by macrophages, which may be a major source of locally-produced pro-inflammatory cytokines [63,64]. TNF-α and other pro-inflammatory molecules in WAT have been implicated in the development and maintenance of obesity-induced adipose tissue inflammation [62]. TNF-α is overproduced in the WAT of several animal models of obesity. Furthermore, macrophage-specific disruption of the NF-κB pathway resulted in improved insulin sensitivity [65]. Ablation of JNK1 in hematopoietically-derived cells including macrophages also protected mice from diet-induced inflammation and insulin resistance without affecting adiposity [66]. These data collectively demonstrate that macrophage inflammation is an important mediator of obesity-induced insulin resistance. Interestingly, weight loss is associated with a reduction in the macrophage infiltration of WAT and an improvement of the inflammatory profile of gene expression.

The cholinergic anti-inflammatory pathway has been extensively studied in terms of its immunomodulatory function against chronic inflammatory disorders [67,68]. Recent studies showed that activation of the cholinergic anti-inflammatory pathway ameliorates obesity-induced inflammation and insulin resistance [7]. Activation of the cholinergic anti-inflammatory pathway by low-dose nicotine significantly suppressed inflammation in adipose tissue, an important site in mediating obesity-induced inflammation in genetically obese (*db/db*) and diet-induced obese (DIO) mice. This was associated with a significant improvement in glucose homeostasis and insulin sensitivity without changes in body weight. In addition, macrophages isolated from mice deficient in α7nAChR had elevated pro-inflammatory cytokine production in response to free fatty acids and TNF-α, known agents causing inflammation and insulin resistance. Furthermore, nicotine significantly suppressed TNF-α-induced cytokine production in wild type, but not α7nAChR -/- macrophages [7]. Overall, these findings suggest that nicotine and specific α7nAChR agonists may be beneficial in the prevention and treatment of obesity-induced inflammation and insulin resistance. However, there is also evidence that heavy smoking affects body fat distribution that is associated with central obesity and insulin resistance [69]. Moreover, smoking appears to aggravate insulin resistance in persons with type 2 diabetes and to impair glycemic control [70]. Other factors such as low physical activity and poor diet could counterbalance and even overtake the slimming effect of smoking. Clearly, the pathophysiological factors involved in the association among smoking and obesity are little explored, and remain to be elucidated.

### Nicotine alleviates ulcerative colitis

One of the earliest noted effects of nicotine on a peripheral tissue was in inflammation of the intestine. Early reports mentioned patients with ulcerative colitis who upon cessation of smoking experienced more severe disease progression, which was ameliorated by returning to smoking [71-73]. In contrast, patients with Crohn's disease experienced severe disease when smoking, requiring the immediate cessation of any tobacco product use [74]. Crohn's disease is a chronic inflammatory disease, which might affect any part of the GI tract, causing a wide range of complications including ulceration, fibrostenosis, and fistula development resulting in symptoms like abdominal pain, fever, diarrhea, and weight loss during episodes with flare-ups. Smoking also worsens the course of Crohn's disease by increasing the risk of developing fistulas and strictures as well as accelerating the need for surgery, probably due to an increased influx of neutrophils into the intestinal mucosa [75,76]. These detrimental effects of smoking in Crohn's disease could also be related to the nicotine-induced suppression of antimicrobial activity and immune responses by macrophages [77], which might further compound any deficiency in the host response to luminal bacteria.

Ulcerative colitis is a chronic IBD characterized by pathological mucosal damage and ulceration, which usually is limited to the rectum (40%) or distal colon (40%) [78]. Patients with ulcerative colitis have increased intestinal permeability, which is most likely caused by the ulcerations observed in ulcerative colitis, causing diarrhea, a primary exudate of the disease [79]. The annual incidence of ulcerative colitis in the United States during the period 1996-2002 was 12 cases per 100,000 and has risen in recent decades [80]. Ulcerative colitis typically presents as a relapsing disorder marked by attacks of diarrhea containing blood and mucus that sometimes persists for months only to recur after an asymptomatic interval of months to years. During relapses, acute attacks of ulcerative colitis cause a massive infiltration of neutrophils and mononuclear cells into the lamina propria and submucosa. During remissions of active disease, granulation tissues fill the ulcer craters accompanied by regeneration of the mucosal epithelium [78].

The recommended first-line therapy of colitis is the anti-inflammatory agent 5-aminosalicytic acid (5-ASA; mesalamine), which targets peroxisome proliferator-activator receptor-γ (PPAR-γ). PPAR-γ is known to be involved in ulcerative inflammation; however, independent actions of 5-ASA include the inhibition of prostaglandin synthesis and NF-κB). 5-ASA may also act as an antioxidant by scavenging oxygen free radicals. In addition to 5-ASA, nicotine has been found to alleviate ulcerative colitis [81]. In fact, ulcerative colitis is largely a disease of non-smokers and ex-smokers, and is uncommon amongst smokers. Although the effects of "smoking" should not be considered synonymous with "nicotine", there is clinical evidence to suggest that nicotine is responsible for this effect, as transdermal nicotine has been used with beneficial effects in patients with active disease [8]. A nicotine enema has also been developed and found to be of benefit when given as additional therapy in active distal ulcerative colitis [82]. Although the specific mechanisms underlying this effect remain unclear, nicotine has a number of actions that could be potentially beneficial, including effects on the immune system [83,84] and gut motility [85].

### Increased severity of colitis in mice deficient in α7nAChR

A major role of α7nAChR in colitis was demonstrated by the increased severity of colitis induced by dextran sulfate sodium (DSS) in α7nAChR-deficient mice. α7nAChR-deficient mice lost significantly more body weight and had increased levels of proinflammatory cytokines in comparison to wild type mice as early as 3 days post-colitis [86]. In addition, neither nicotine nor a selective α7nAChR agonist (choline chloride) attenuated the degree of inflammation in α7nAChR-deficient mice. Nicotine has been found to reduce the LPS-stimulated production of TNF-α and IL-1β from peripheral blood mononuclear cells from IBD patients [87]. Thus, it is not surprising that excessive TNF-α production as occurs in colitis can also be attenuated by activation of α7nAChR [86].

Macrophages are an important component of the inflammatory response in murine models of colitis and in human IBD and are responsible for the production of pro-inflammatory cytokines. Several groups have identified the α7nAChR on macrophages suggesting that nicotine modulates the activity of these cells. However, several immune cells (e.g., dendritic cells, mast cells) express α7nAChR and other nAChR subtypes suggesting that different types of immune cells are sensitive to acetylcholine. An interesting issue to be addressed is which nAChRs, or their respective levels of expression, might participate in colitis and the differential response to nicotine. In fact, very little is known about the signaling pathways activated by nicotine or the mechanism mediating nicotine-associated anti-inflammation in the bowel. An immune regulating role for the cholinergic nervous system may be particularly evident in intestinal tissue, given the dense cholinergic innervation and the abundant number of resident macrophages that populate the intestinal mucosa and muscularis externa, of which some are closely associated with cholinergic fibers.

In isolated intestinal and peritoneal macrophages, nAChR activation enhanced endocytosis and phagocytosis and this effect induced a transiently enhanced mucosal passage of luminal bacteria, in agreement with the role of ACh in stress-induced epithelial permeability [88]. The effect was mediated via stimulated recruitment of GTPase Dynamin-2 to the forming phagocytic cup and involved nAChR α4/β2, rather than α7nAChR. However, despite enhanced luminal bacterial uptake, ACh reduced NF-κB activation and pro-inflammatory cytokine production, while stimulating anti-inflammatory interleukin-10 production [89].

### α7nAChR agonists worsen colitis

Given the proposed role of the α7nAChR in mediating the effects of stimulation of cholinergic anti-inflammatory pathways, selective α7nAChR agonists may have more therapeutic potential in ameliorating colitis than nicotine. Snoek et al. [90] explored the effects of nicotine and two selective α7nAChR agonists (AR-R17779, GSK1345038A) on disease severity in two mouse models of acute experimental colitis. Colitis was induced by administration of DSS (1.5%) in the drinking water or 2,4,6-trinitrobenzene sulphonic acid (TNBS; 2 mg) intrarectally. Nicotine, AR-R17779, or GSK1345038A was administered daily by i.p. injection. After 7 days clinical parameters and colonic inflammation were scored.

Nicotine and both α7nAChR agonists reduced the activation of NF-κB and pro-inflammatory mediator release in whole blood and macrophage cultures. In addition, treatment of DSS colitis with nicotine led to a significant reduction in colonic edema and colonic IL-6 and IL-17 production. However, this reduction was not marked enough to be reflected in clinical parameters and histopathological scores. Treatment with the α7nAChR agonists both displayed a bell-shaped dose-response curve; the highest doses of AR-R17779 and GSK1345038A significantly ameliorated clinical parameters, whereas lower doses of both compounds actually worsened or did not affect clinical parameters. It should be borne in mind that several nAChRs are expressed in the gut and on various cell types. Intestinal mucosal macrophages express α4β2 nAChR and assist in the surveillance of luminal antigen uptake by augmenting the uptake of luminal bacteria by mucosal macrophages. Previous studies also point towards a role in modulation of intestinal inflammation by the α5nAChR [91](see Below). Thus, a combination of selective α7nAChR, α4β2 nAChR and/or α5nAChR agonists might be more appropriate in modulating intestinal inflammation as a large array of AChRs are expressed in the gut. Irrespectively, nicotine administration ameliorated disease in previous studies of experimental colitis [37].

### Dysfunction of Enteric Neural Circuits in Colitis

**Figure 2:**
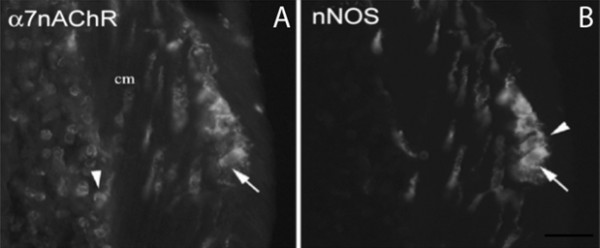
**Immunohistochemical localization of α7nAChR in the murine enteric nervous system**. A. Confocal image of a cryostat section of the colon stained using a polyclonal antibody raised against the alpha bungarotoxin-sensitive receptor subunit α7nAChR (1:50; Abcam). The specificity of the antibody was confirmed by Western blot and demonstrating α7nAChR immunoreactivity in the adrenal medulla. B. The same section stained using an antibody to neuronal nitric oxide synthase (nNOS; Upstate Biotechnology). All α7nAChR-positive neurons express nNOS (arrow), a marker of inhibitory motorneurons in the murine colon; however, a subset of nNOS-positive neurons does not express α7nAChR (B; arrowhead). α7nAChR immunoreactivity is also displayed by immune cells in the mucosa (arrowhead). Unpublished research.

In addition to immune cells, neurons in the ENS express α7nAChRs (see Figure 2; [32]). The ENS consists of the intrinsic innervation of the bowel, a component of the autonomic nervous system with the unique ability to function independently from the CNS (for review, see [49]). Enteric ganglia are organized into two major ganglionated plexuses, namely the myenteric (Auerbach's) and submucosal (Meissner's) plexus, and contain a variety of functionally distinct neurons, including primary afferent neurons, interneurons, and motor neurons, synaptically linked to each other in microcircuits. While the myenteric plexus mainly regulates intestinal motility, the submucosal plexus together with nerve fibers in the lamina propria are involved in regulating epithelial transport. These nerves form networks within the lamina propria of both crypts and villi with the terminal axons in close contact with the basal lamina, an ideal position not only to affect epithelial cell functions but also to detect absorbed nutrients and antigens. Substances released from epithelial cells may act on nerve terminals to change the properties of enteric neurons and cause peripheral sensitization. Consequently, permanent or even transient structural alterations in the ENS disrupt normal GI function. Since the ENS controls the motility and secretion of the bowel these abnormalities indicate the impact of inflammation on neural signaling in the ENS.

Several studies have demonstrated structural changes within the ENS in gut inflammation (see [92] for review). For example, damage to axons has been observed in the inflamed human intestine in episodes of IBD [93]. Other changes that occur in the ENS during inflammation include altered neurotransmitter synthesis, content, and release, changes in glial cell numbers and a myenteric ganglionitis associated with infiltrates of lymphocytes, plasma cells and mast cells [94,95]. In fact, consequences of intestinal inflammation, even if mild, persist for weeks beyond the point at which detectable inflammation has subsided [92].

To identify cells through which nicotine might exert its beneficial effects in colitis, we localized α7nAChR in the guinea-pig colon [32] and more recently, in the murine colon (Figure 2) utilizing a polyclonal antibody to α7nAChR (1:50; Abcam). The specificity of the antibody was confirmed by Western blots and demonstrating α7nAChR immunoreactivity in the adrenal medulla. Immunohistochemistry localized α7nAChR protein to cells in the mucosa and enteric neurons. All α7nAChR-positive neurons in the myenteric plexus contained nitric oxide synthase (NOS) a marker of inhibitory motorneurons in the mouse colon. Numerous α7nAChR-ir nerve fibers were present in the circular muscle layer. Animal studies have shown that nicotine produces smooth muscle relaxation largely through the release of NO. This action of nicotine has been confirmed in the human sigmoid colon and could account for rapid and dramatic relief of fecal urgency and frequency reported by some ulcerative colitis patients given nicotine [11].

**Figure 3:**
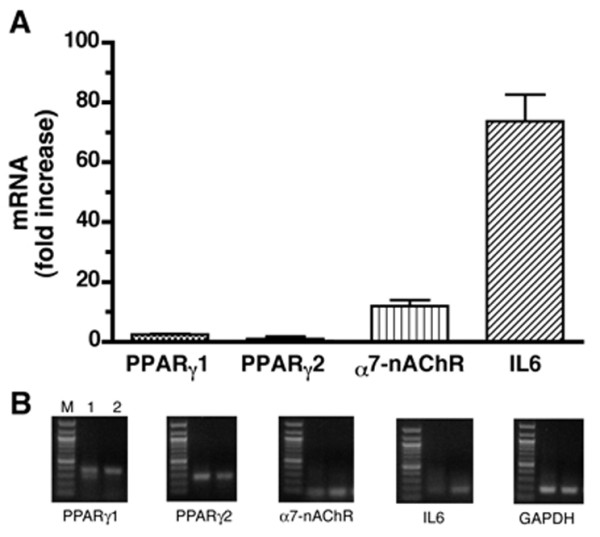
**Inflammation up-regulates the expression of α7nAChR mRNA in the murine colon**. A. Samples of colon were collected at 7 days from dextran sulfate sodium (DSS)-treated (n = 5) and control (n = 5) mice and expression of α7nAChR mRNA was determined in preparations of longitudinal muscle with adherent myenteric plexus (LMMP) using real-time reverse transcriptase polymerase chain reaction (RT-PCR). RT-PCR demonstrated α7nAChR mRNA in the LMMP and an increase in its expression by inflammation. Significant up-regulation of IL6 mRNA was also observed while proliferator-activated receptor-γ (PPAR-γ)1 and PPAR-γ2 mRNA levels remained unchanged. B. RT-PCR analysis of α7nAChR, IL6, PPAR-γ1 and PPAR-γ2 mRNA expression in LMMP isolated from the normal and inflamed murine colon. M = markers; Lane 1 = control; Lane 2 = inflamed colon. Unpublished research.

Little is known about the significance of enteric nAChRs in inflammation or the function of α7nAChR in particular. To confirm α7nAChR expression in the ENS and determine whether inflammation can affect α7nAChR expression in the gut we isolated the longitudinal muscle with adherent myenteric plexus (LMMP) from the inflamed colon of DSS-treated (n = 5) and control (n = 5) mice and α7nAChR expression was analyzed using real-time reverse transcriptase polymerase chain reaction (RT-PCR). The level of α7nAChR mRNA expression in the LMMP was significantly (*p *< 0.05) increased in colitis (See Figure 3) demonstrating that inflammation can modulate α7nAChR expression and signaling in the ENS. A well-documented and significant up-regulation of IL6 mRNA expression was also observed while the expression of PPAR-γ1 and PPAR-γ2 remained unchanged (Figure 3). These findings confirm α7nAChR expression in the ENS and a putative role in gut inflammation.

### Other nAChRs in colitis

Although a great deal of attention has been given to α7nAChR in peripheral disease and inflammation, it is premature to assume that this receptor is alone in its participation in modulating the peripheral inflammatory status. In fact, nAChR subunit mRNA for α3, α5, β2, and β4 has been detected in multiple cell types of the intestine suggesting that, as in the brain, nicotine may impact upon different inflammatory processes with considerable specificity depending upon the nAChR subtypes present. Xu et al. [96] reported that mice lacking α3nAChR or both nAChRβ2 and nAChRβ4 have similar autonomic dysfunction of the bowel. Studies by [91] demonstrated that the α5nAChR might participate in colitis and the differential response to nicotine. Mice deficient in α5nAChR are more susceptible to experimentally induced colitis than their wild-type controls. However, transdermal nicotine attenuated the disease process in both wild type and knockout mice, although to a greater extent in the knockout mice, suggesting that the absence of α5nAChR increases the susceptibility to disease initiation and the presence of α5nAChR in the wild-type animal appears to enhance the therapeutic sensitivity to nicotine.

## Conclusion

Much work remains in terms of understanding the anti-inflammatory effects of nicotine in obesity-related inflammation and ulcerative colitis. However, it is now known that the α7nAChR plays a major role in the anti-inflammatory effects of nicotine and nicotine attenuates inflammation in both obesity and ulcerative colitis. What these findings suggest is the potential use of selective α7nAChR agonists as a new class of anti-inflammatory drugs. Despite tremendous efforts, obesity and obesity-related disorders remain at epidemic proportions and the etiology of ulcerative colitis remains unclear. Since the inflammatory response is an integral process in both obesity and ulcerative colitis, controlling the inflammatory response could ameliorate tissue damage. The effectiveness of α7nAChR agonists as a drug target will ultimately depend upon a clear understanding of the collective biological consequences of peripheral nAChR expression on inflammation. In addition, it should also be considered that the development of nicotine as a therapeutic intervention has its limitations due to toxicity related side effects and pharmacological non-specificity.

## Abbreviations

5-ASA: 5-aminosalicytic acid; ARE: AU-rich element; ACh: acetylcholine; BTX: bungarotoxin; CLP: cecal ligation and puncture; DSS: dextran sulfate sodium; ENS: enteric nervous system; GI: gastrointestinal; HMGB1: high mobility group box 1; IBD: inflammatory bowel disease; IKKβ: inhibitor of NF-κB kinase β; IL: interleukin; JNK: c-Jun NH_2_-terminal kinase; LMMP: longitudinal muscle with adherent myenteric plexus; LPS: lipopolysaccharide; nNOS: neuronal nitric oxide synthase; NOS: nitric oxide synthase; nAChR: nicotinic acetylcholine receptor; NF-kB: nuclear factor kappa B; NPY: neuropeptide Y; PPAR-γ: peroxisome proliferator-activator receptor-γ; ROS: reactive oxygen species; RT-PCR: real-time reverse transcriptase polymerase chain reaction; TNBS: 2,4,6-trinitrobenzene sulphonic acid; TNF: tumor necrosis factor; Tregs: CD4(+)CD25(+) regulatory T cells; TTP: tristetrapolin; WAT: white adipose tissue.

## Competing interests

The authors declare that they have no competing interests.

## Authors' contributions

All authors participated in the preparation of the manuscript, and read and approved the final manuscript.
